# Supplementary material for: O-GlcNAc Transferase Inhibitor Synergistically Enhances Doxorubicin-Induced Apoptosis in HepG2 Cells
Source: Cancers (Basel). 2020 Oct 27;12(11):3154. doi: 10.3390/cancers12113154 (PMC7693581; doi:10.3390/cancers12113154)
Supplement: Supplementary file 1 [file cancers-12-03154-s001.pdf]

Supplementary Materials: O-GlcNAc Transferase Inhibitor Synergistically Enhances Doxorubicin-Induced Apoptosis in HepG2 Cells

Su Jin Lee and Oh-Shin Kwon

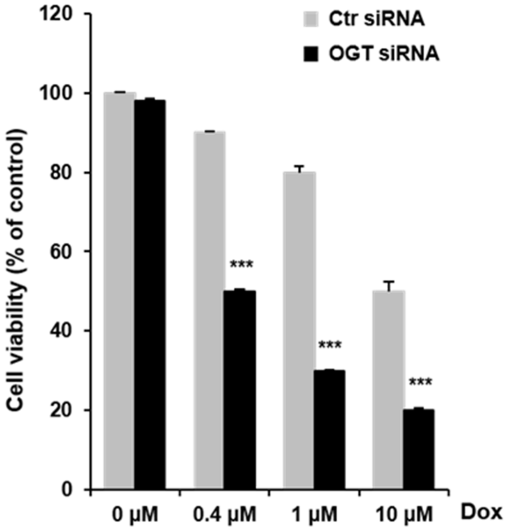

Figure S1. Effects of OGT siRNA and DOX on viability of HepG2 cells. After transfection with control siRNA or OGT siRNA, HepG2 cells were treated with DOX (0.4, 1, or 10 μM) or without DOX for 15 h. Cell viability was analyzed by MTT assay. ****p* < 0.005 compared with control siRNA group.

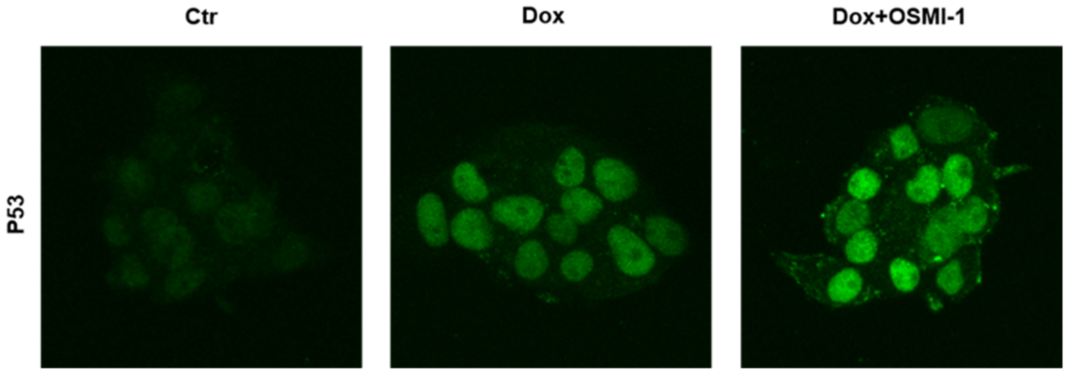

Figure S2. OSMI-1 enhances DOX-induced expression of P53 in HepG2 cells. HepG2 cell were grown on 8 well chamber slides. Cells were pretreated with DOX or OSMI-1 for 15h. cells were fixed with 4% paraformaldehyde for 10 min, permeabillized with 0.5 % triton X-100 for 10 min, and blocked with 3% BSA for 1h. The cells were incubated with antibody against P53. The cells were visualized by fluorescence microscopy (original magnification, ×200).

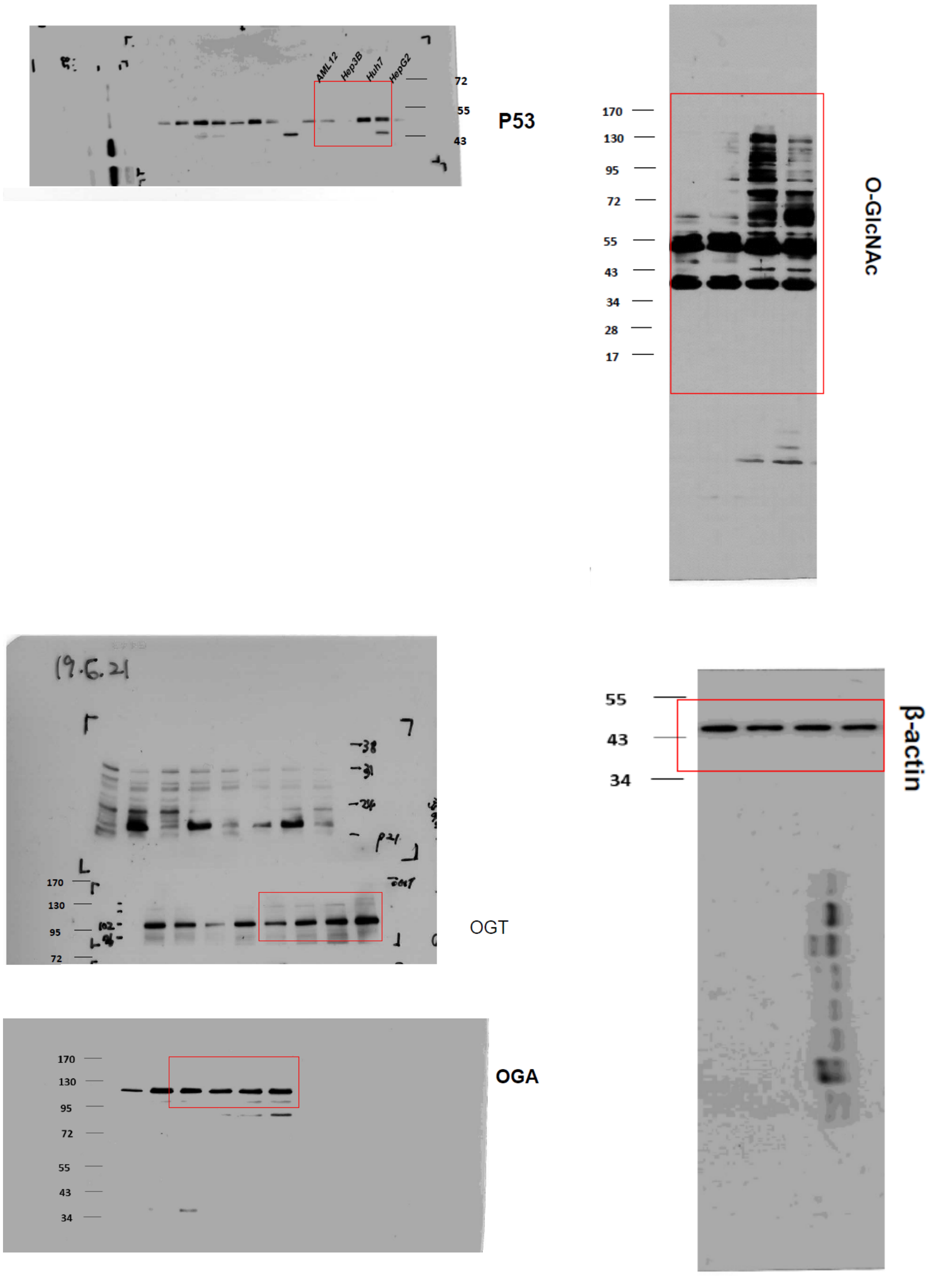

Figure S3. Uncropped western blot figure of Figure 1A.

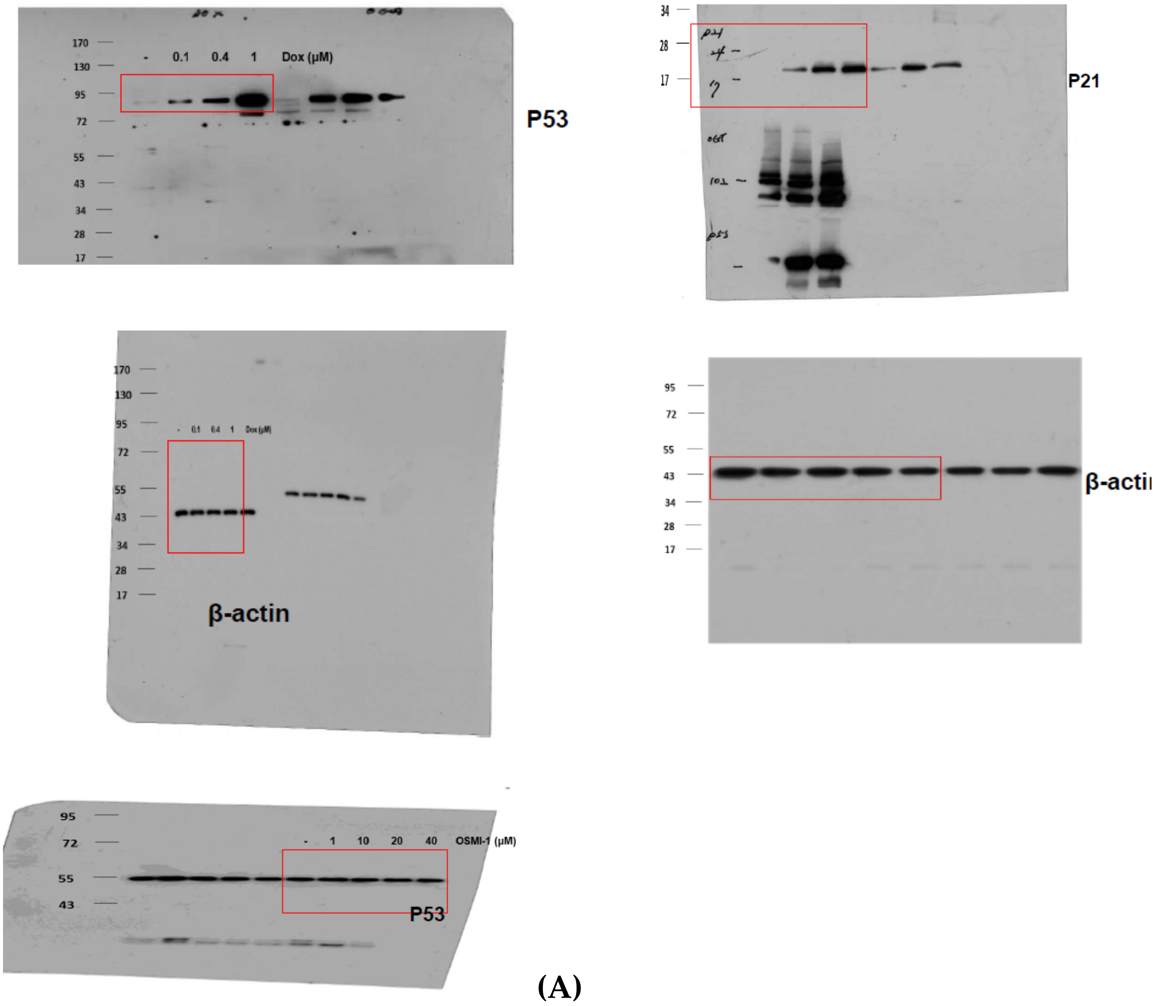
170
130
95
72
55
43
34
28
17
- 0.1 0.4 1 Dox (μM)
P53
P21
β-actin
β-acti
- 1 10 20 40 OSMI-1 (μM)

(A)

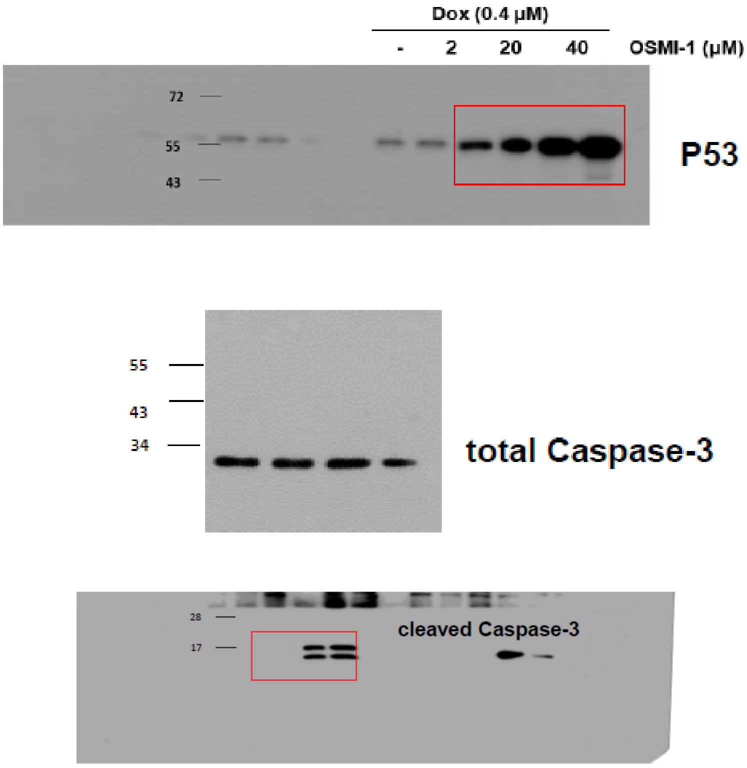
Dox (0.4 μM)
- 2 20 40 OSMI-1 (μM)
72
55
43
P53
34
total Caspase-3
28
17
cleaved Caspase-3

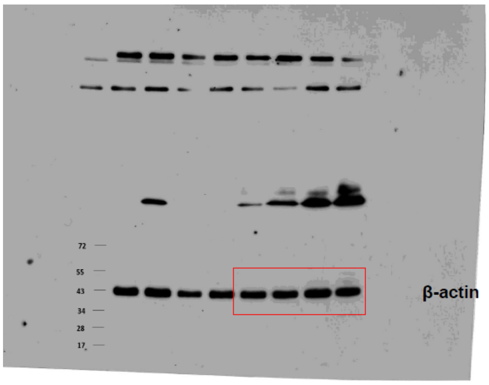
72
55
43
34
28
17
β-actin

(B)

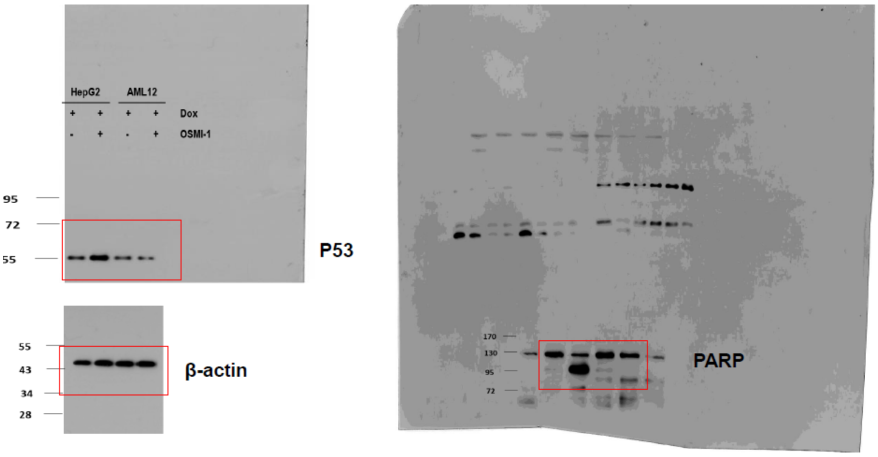
HepG2
AML12
Dox
OSMI-1
95
72
55
P53
55
43
34
28
β-actin
170
130
95
72
PARP

(C)

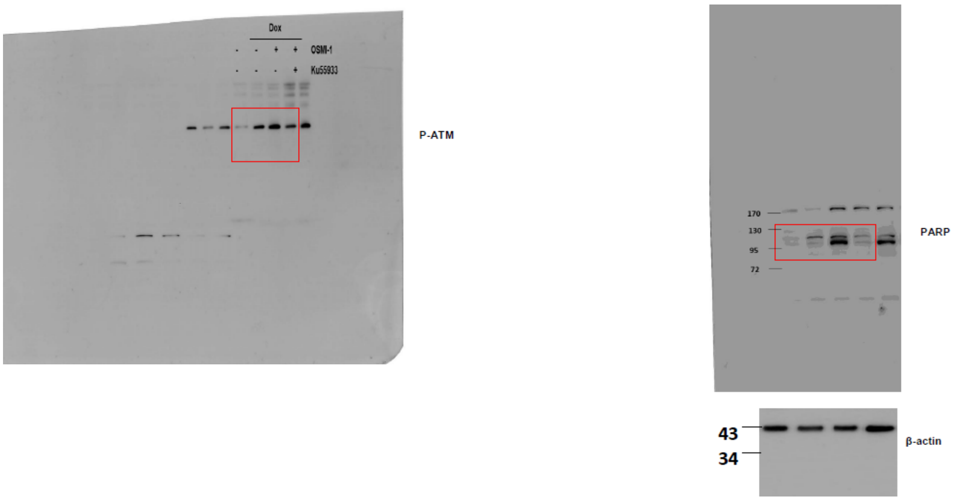
Dox
OSMI-1
Ku55933
P-ATM
170
130
95
72
PARP
43
34
β-actin

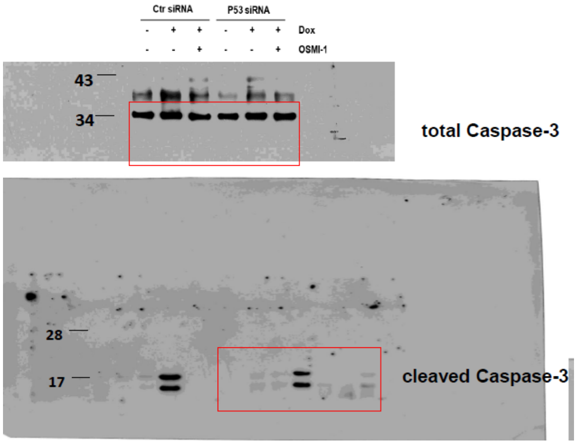
Ctr siRNA
P53 siRNA
Dox
OSMI-1
43
34
total Caspase-3
28
17
cleaved Caspase-3

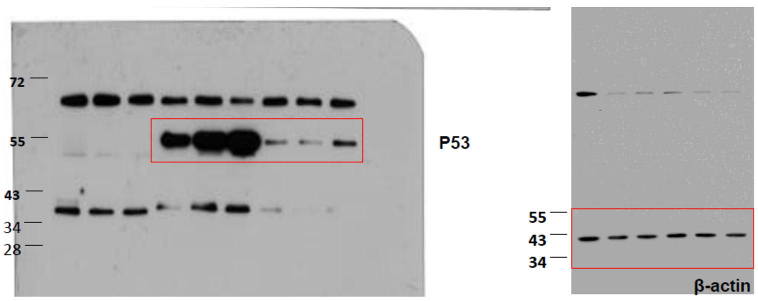

(D)

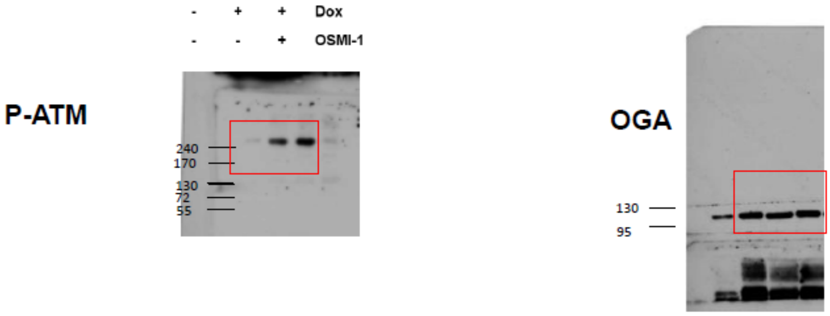

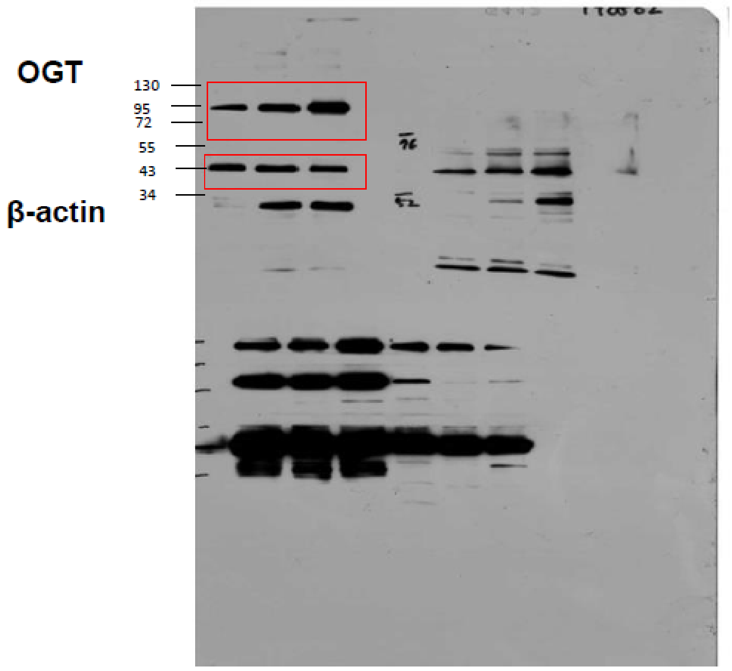

(E)

Figure S4. Uncropped western blot figure of Figure 2 (A–E).

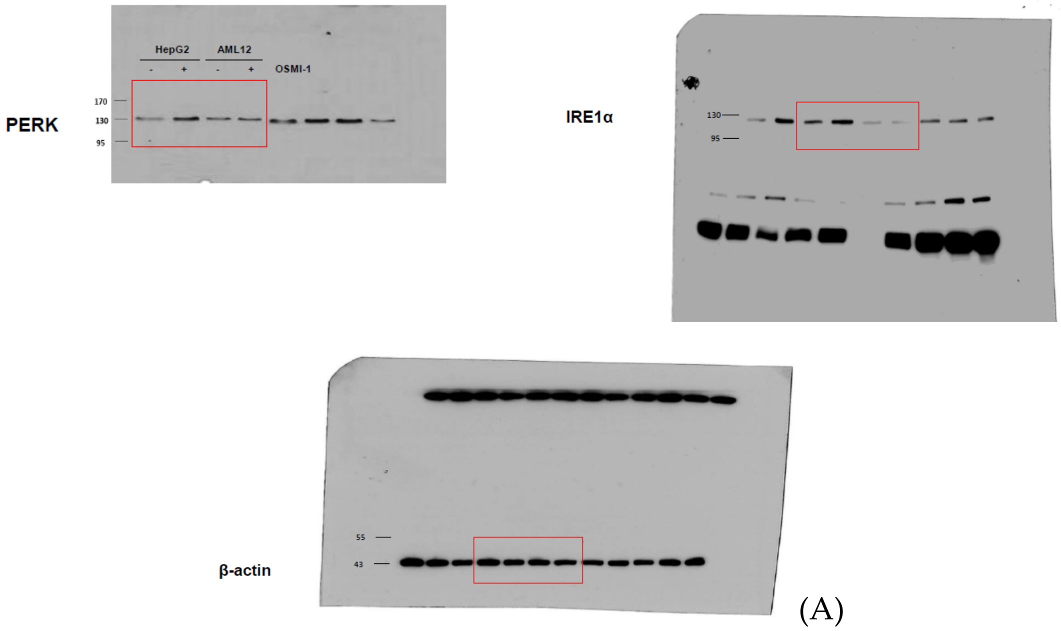
PERK
HepG2
AML12
- + - +
OSMI-1
170
130
95
IRE1α
130
95
β-actin
55
43

(A)

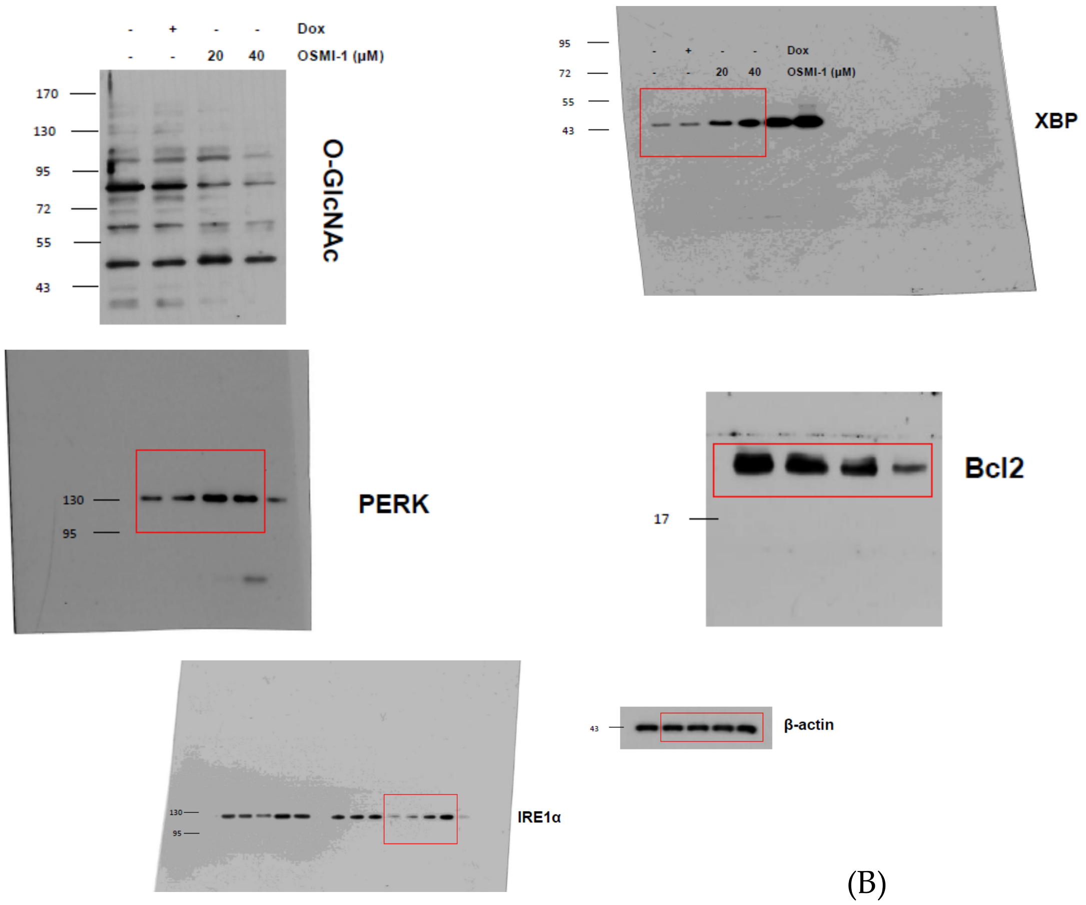
- + - -
Dox
- - 20 40
OSMI-1 (μM)
170
130
95
72
55
43
O-GlcNAc
95
72
55
43
- + - -
Dox
- - 20 40
OSMI-1 (μM)
XBP
130
95
PERK
Bcl2
17
43
β-actin
130
95
IRE1α

(B)

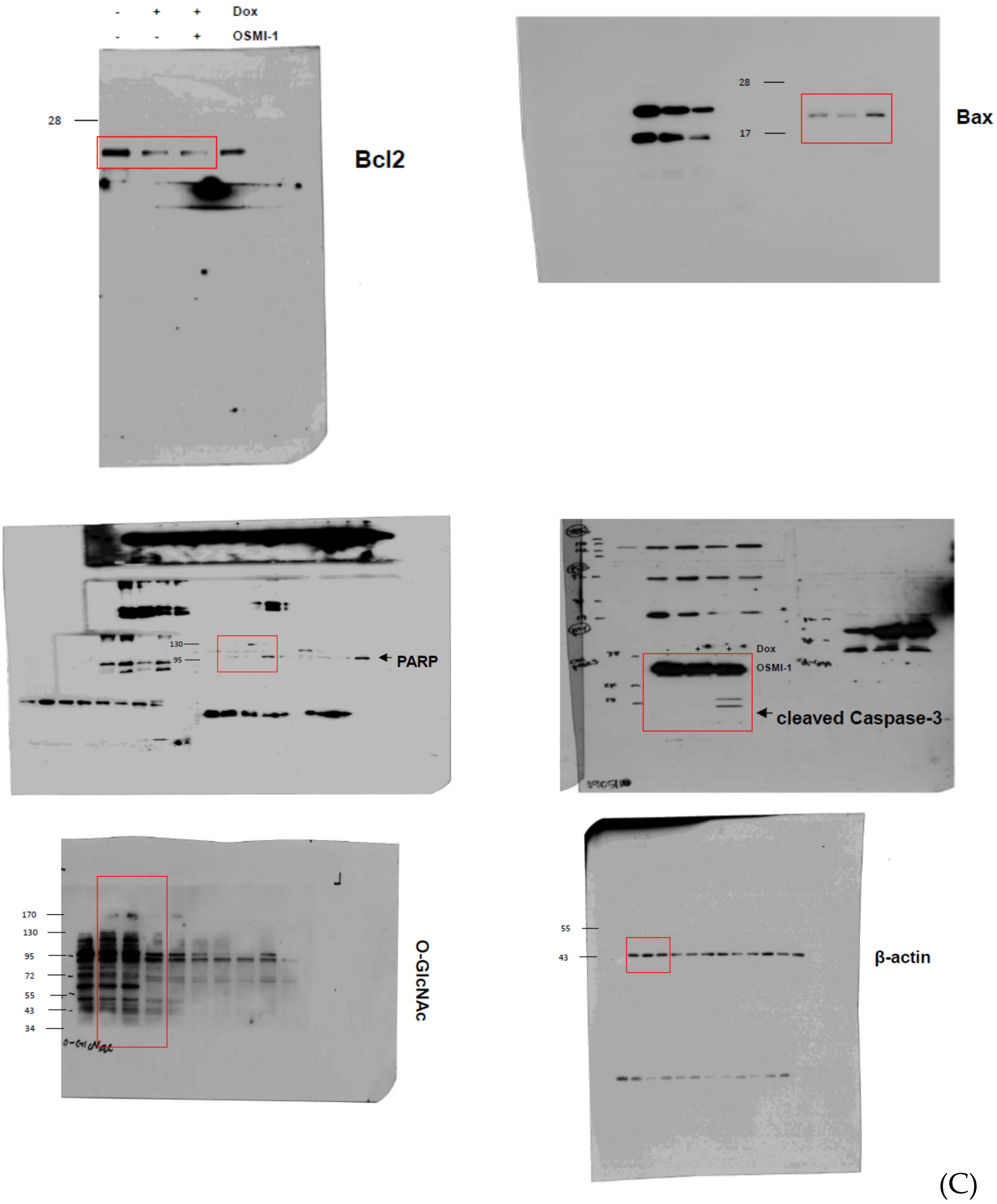

Figure S5. Uncropped western blot figure of Figure 3 (A–C).

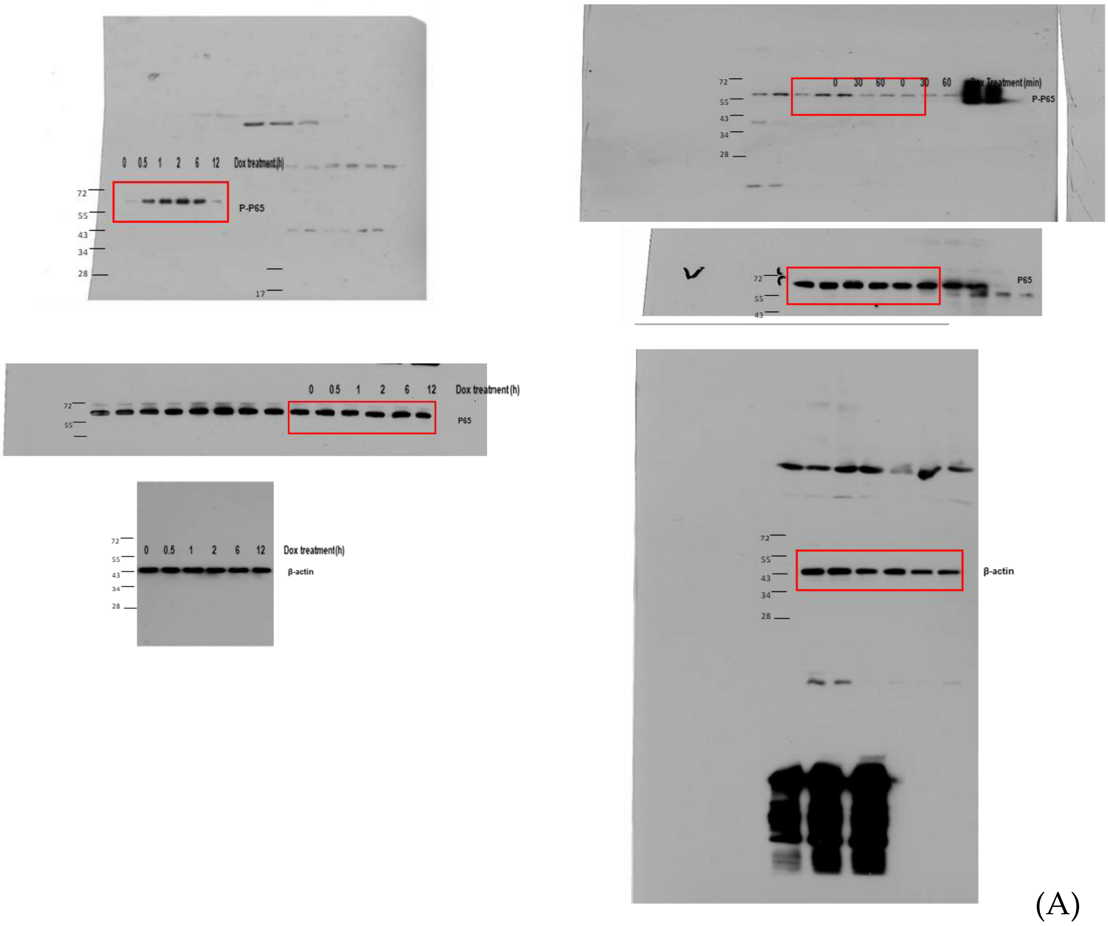
0 0.5 1 2 6 12 Dox treatment(h)
P-P65
72
55
43
34
28
17
P65
β-actin
0 30 60 0 30 60 Dox Treatment (min)

(A)

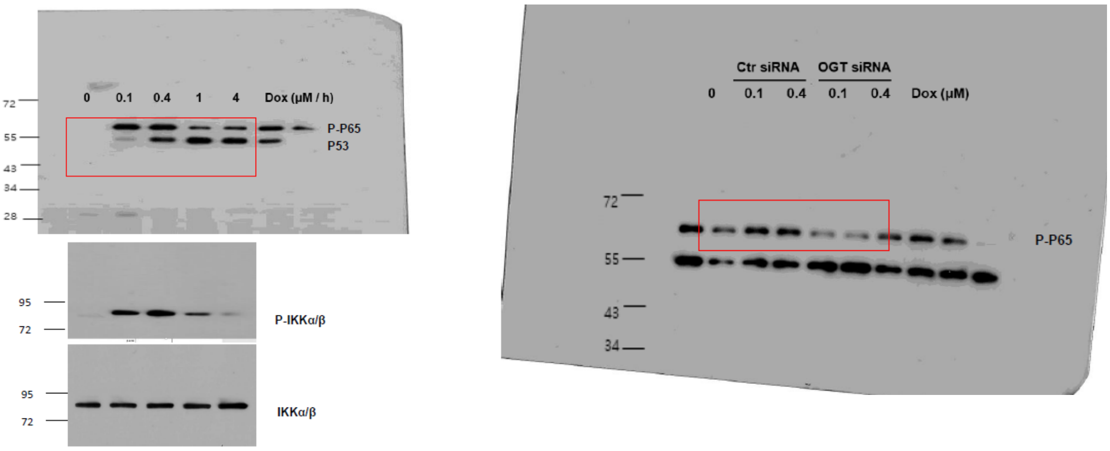
0 0.1 0.4 1 4 Dox (μM / h)
P-P65
P53
P-IKKα/β
IKKα/β
95
72
55
43
34
28
Ctr siRNA
OGT siRNA
0 0.1 0.4 0.1 0.4 Dox (μM)

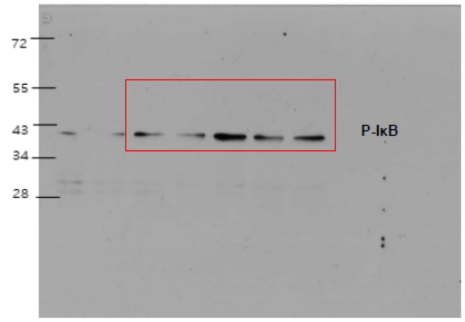
72
55
43
34
28
P-IκB

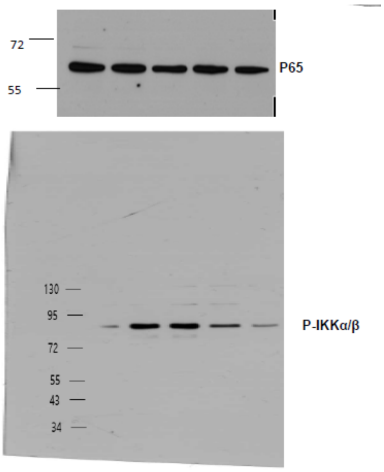
72
55
P65
130
95
72
55
43
34
P-IKKα/β

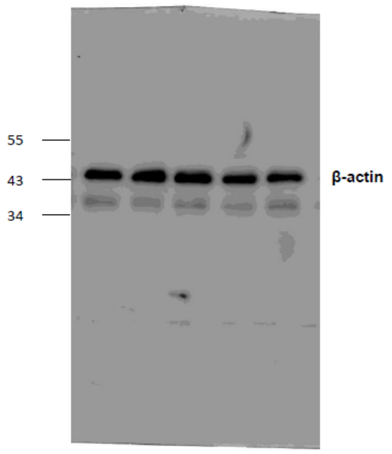
55
43
34
β-actin

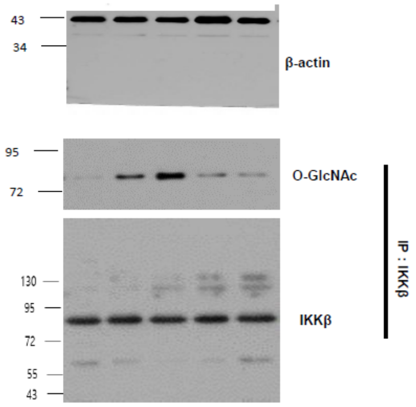
43
34
β-actin
95
72
O-GlcNAc
130
95
72
55
43
IKKβ
IP : IKKβ

(B)

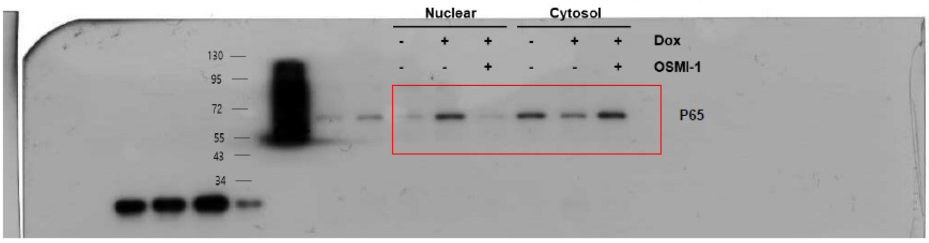
Nuclear
Cytosol
Dox
OSMI-1
130
95
72
55
43
34
P65

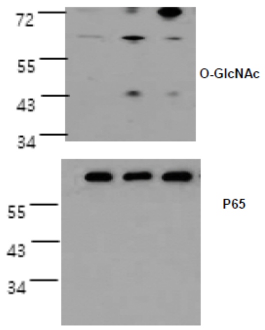
72
55
43
34
O-GlcNAc
55
43
34
P65

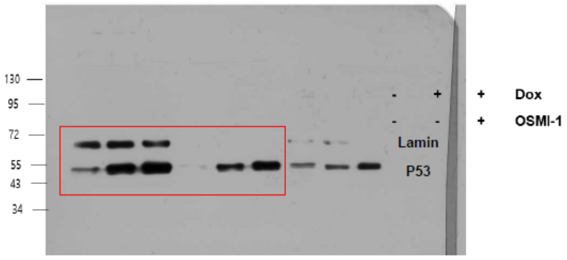
130
95
72
55
43
34
Dox
OSMI-1
Lamin
P53

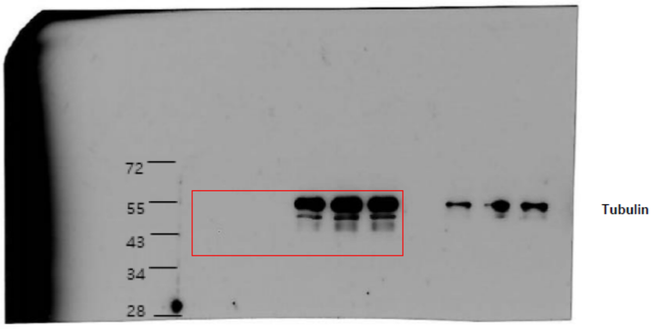

(D)

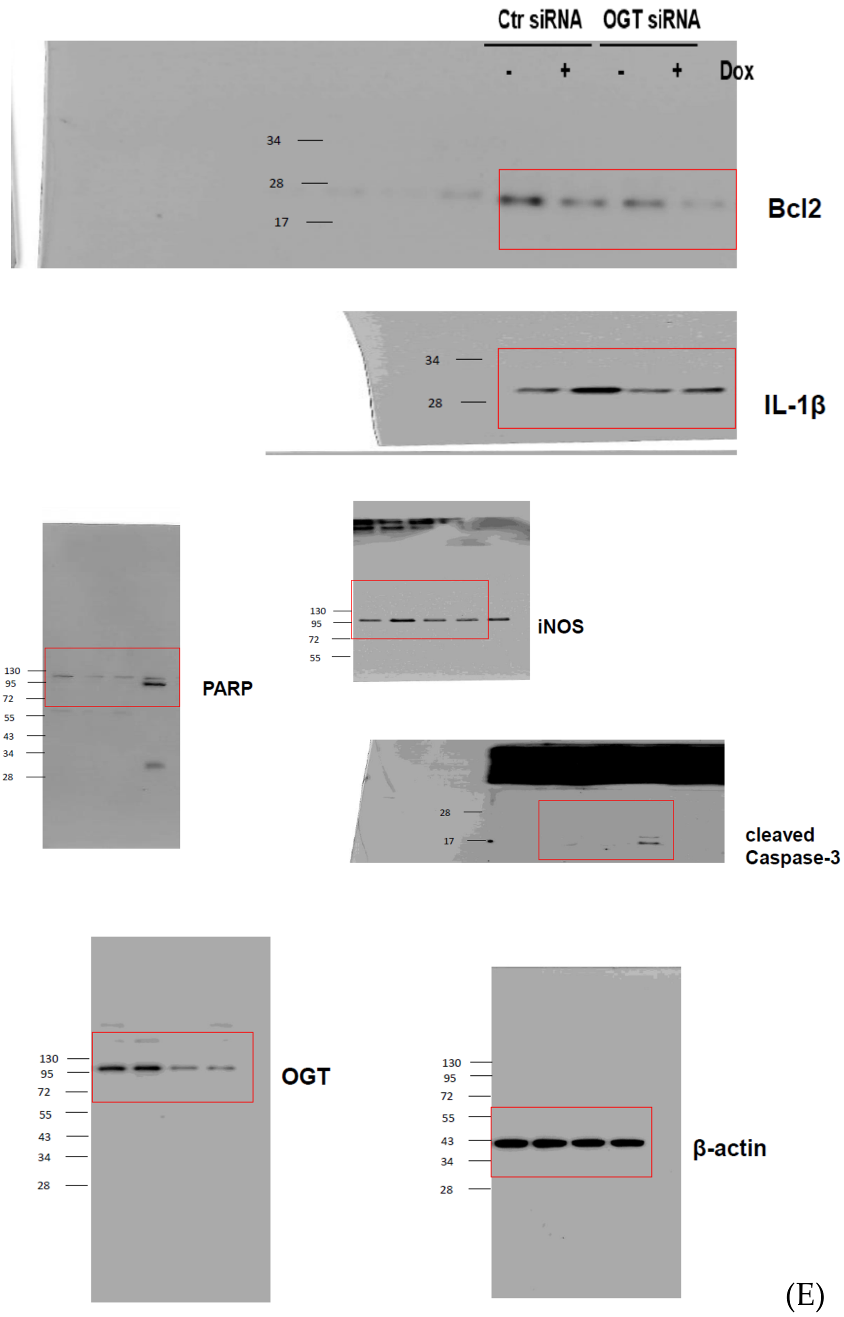

(E)

Figure S6. Uncropped western blot figure of Figure 4A,B,D,E.

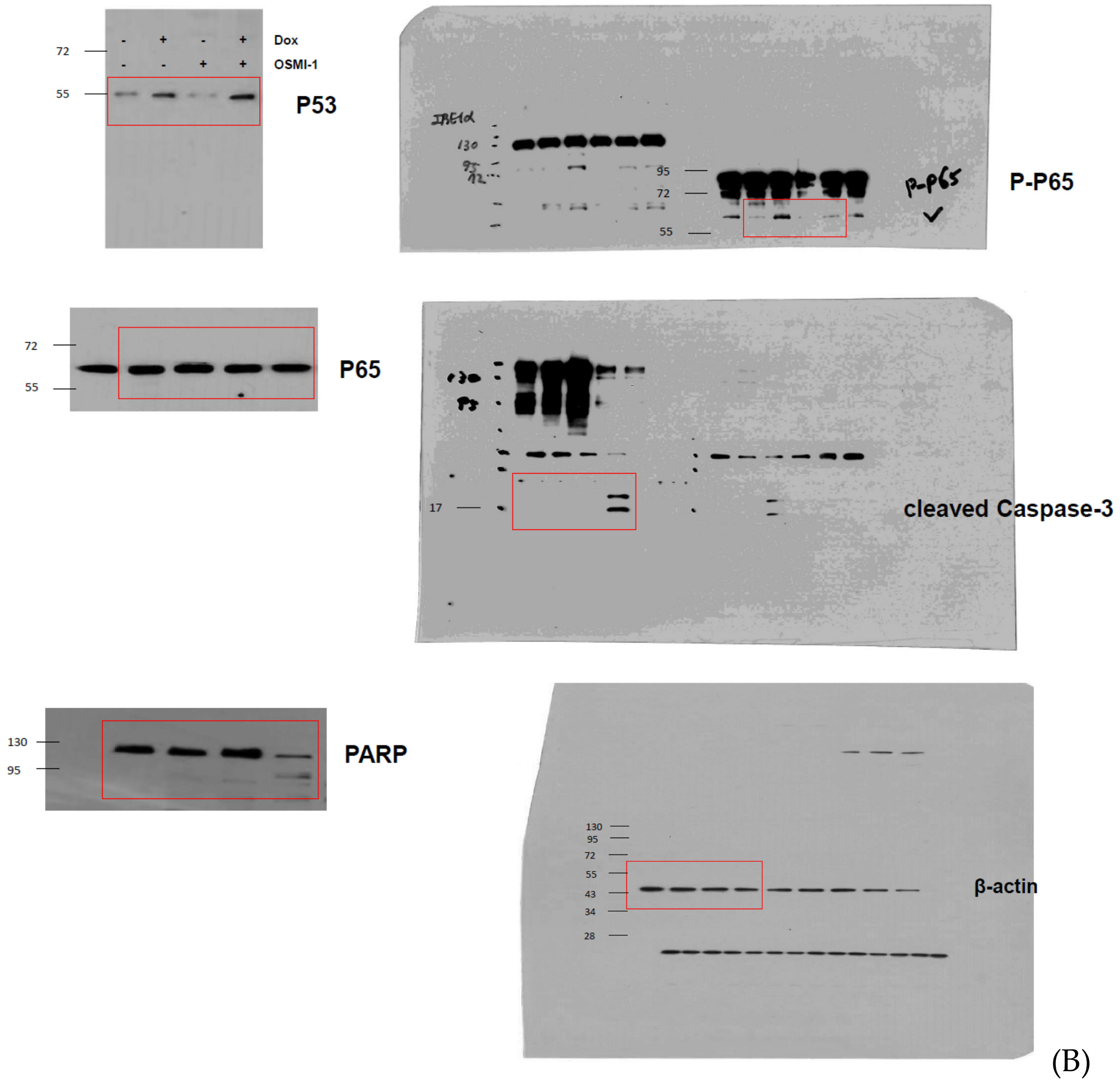
Dox
OSMI-1
72
55
P53
P-P65
95
72
55
P65
72
55
cleaved Caspase-3
17
PARP
130
95
β-actin
130
95
72
55
43
34
28

(B)

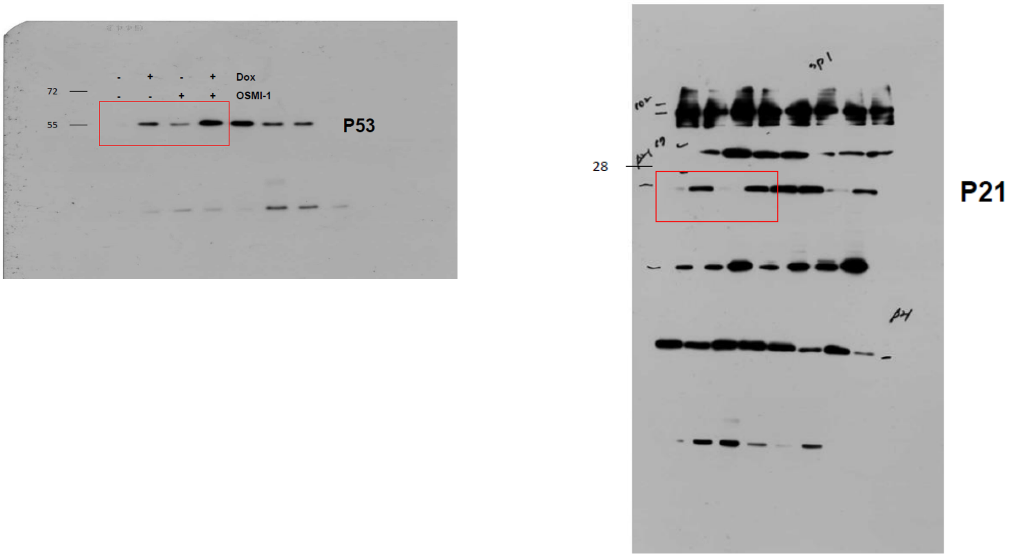
Dox
OSMI-1
72
55
P53
28
P21

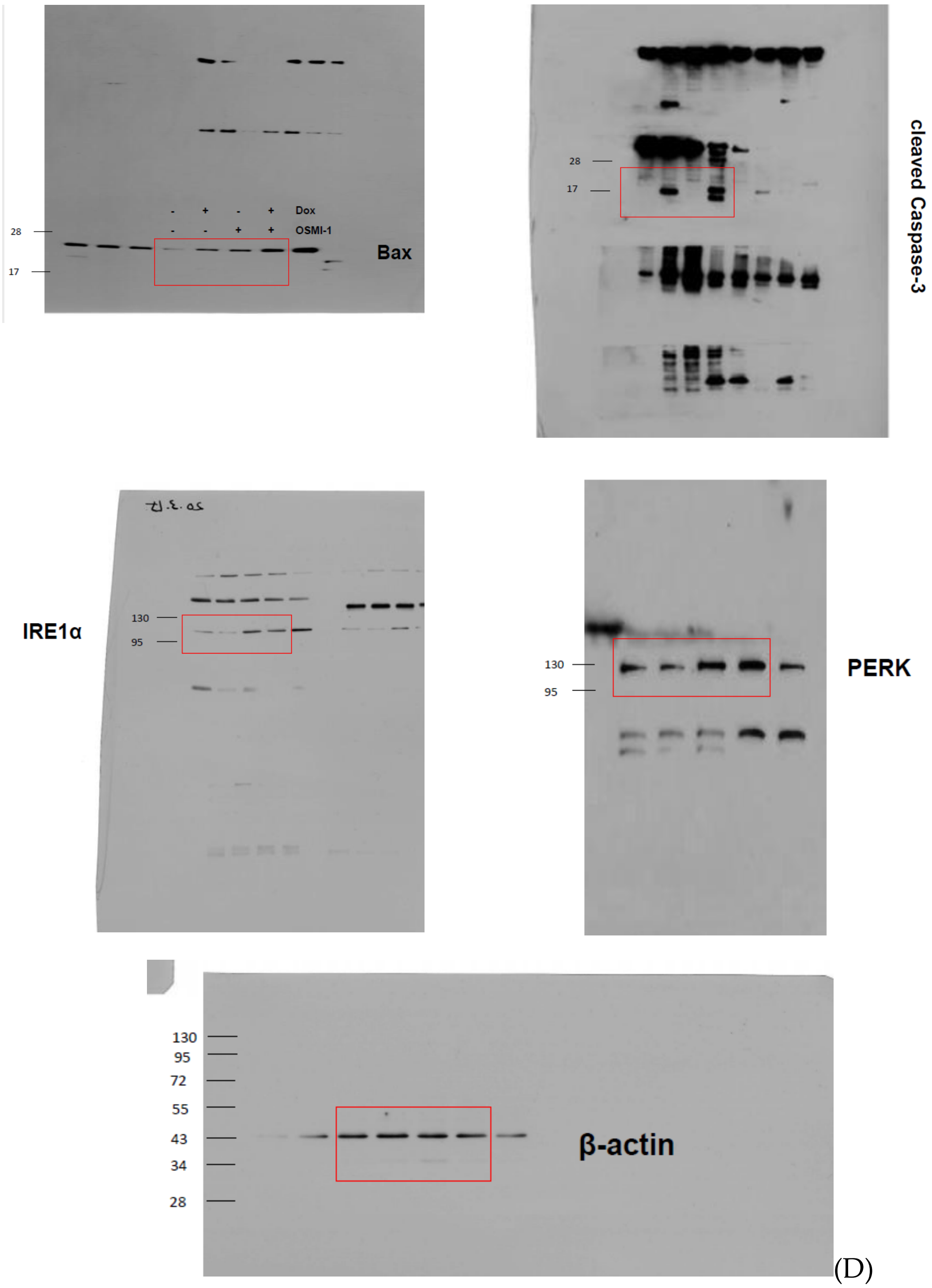

Figure S7. Uncropped western blot figure of Figure 5B,D.

© 2020 by the authors. Submitted for possible open access publication under the terms and conditions of the Creative Commons Attribution (CC BY) license (http://creativecommons.org/licenses/by/4.0/).
